# Supplementary figures and images for: Into the Wild: A novel wild-derived inbred strain resource expands the genomic and phenotypic diversity of laboratory mouse models
Source: PLoS Genet. 2024 Apr 10;20(4):e1011228. doi: 10.1371/journal.pgen.1011228 (PMC11034653; doi:10.1371/journal.pgen.1011228)

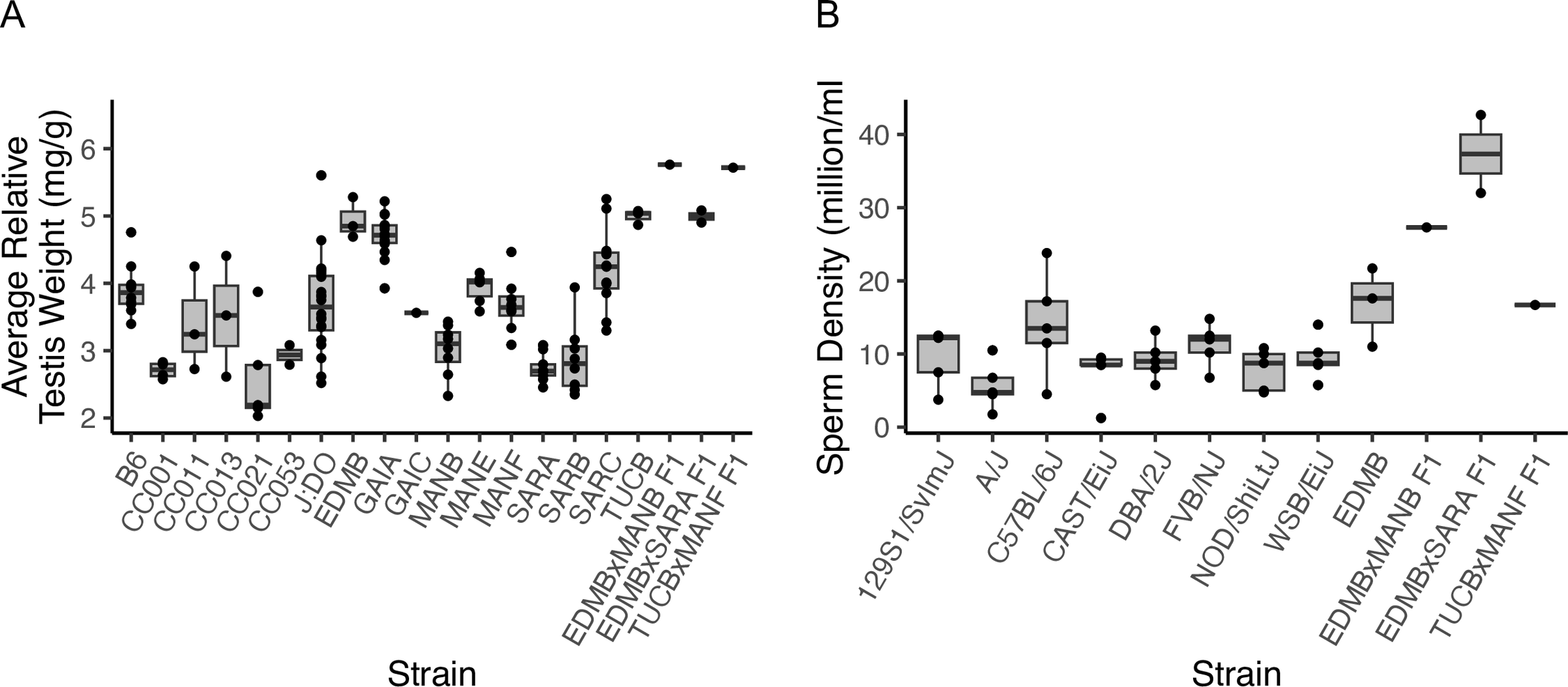

Supplement: S1 Fig — All mice were >8 weeks old at time of phenotyping. (A) Testis weight standardized by body weight for inbred Nachman strains and three derivative F1 hybrids. Values for the classical inbred strain C57BL/6J (B6), a sample of DO animals, and a representative subset of CC strains are included for comparison. (B) Sperm density estimates for 8 genetically diverse inbred mouse strains, EDMB/NachJ (EDMB), and 3 F1 hybrids derived from crosses between Nachman strains. Inbred strain sperm density estimates are from the Handel1 dataset on the Mouse Phenome Database. (TIF) [file pgen.1011228.s001.tif]

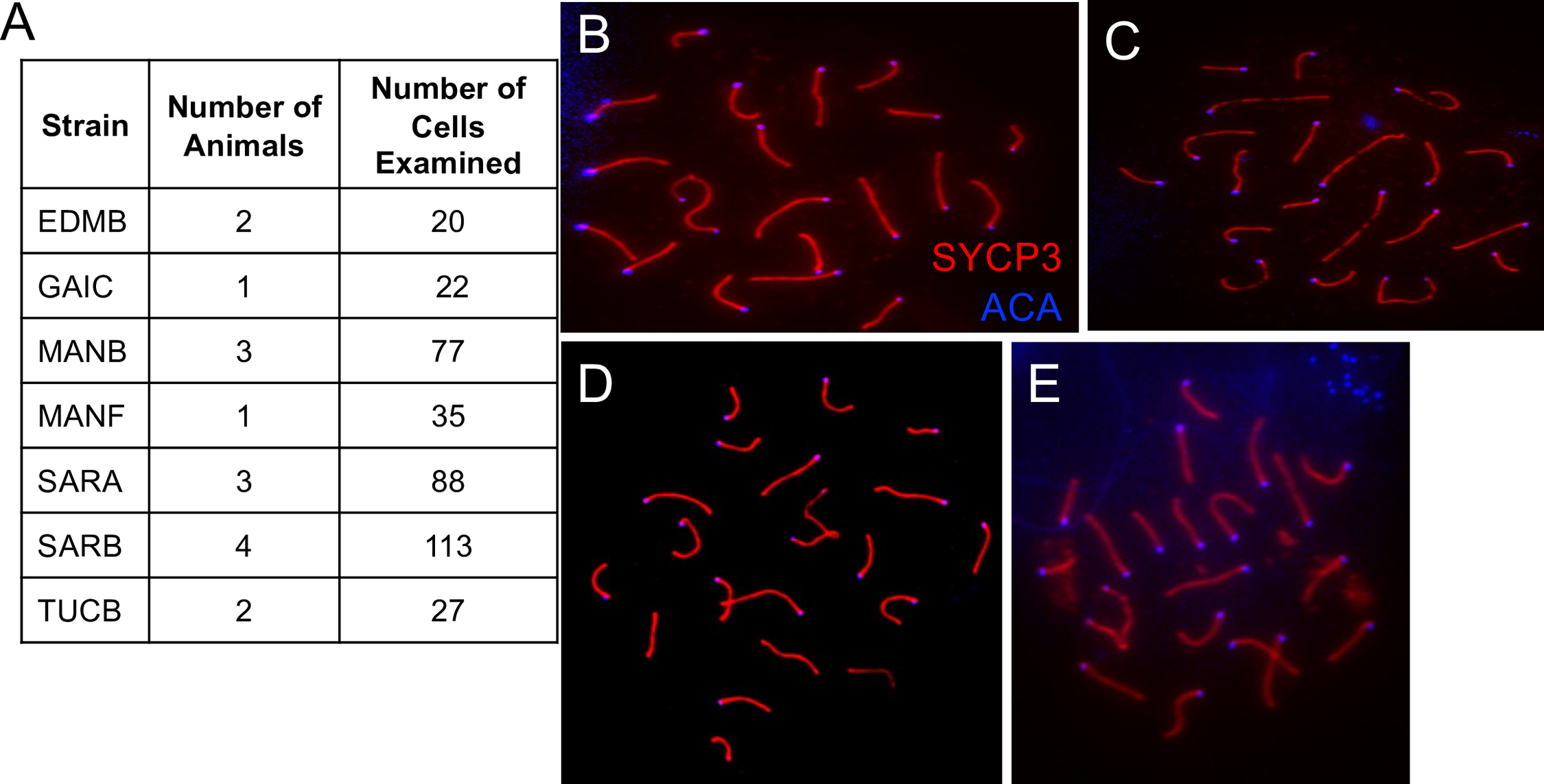

Supplement: S2 Fig — (A) Table summary of the number of individuals and number of cells analyzed for each of 5 Nachman strains. All cells derive from males and all harbor the standard house mouse karyotype defined by 19 acrocentric autosome pairs and a pair of sex chromosomes. Representative pachytene-stage spermatocyte cell spreads from TUCB/NachJ (B) EDMB/NachJ (C), MANF/NachJ (D), and GAIC/NachJ (E) stained with antibodies against SYCP3 (red), a component of the meiotic synaptonemal complex that localizes along the paired chromosome axes, and anti-centromere antibodies (blue). (TIF) [file pgen.1011228.s002.tif]

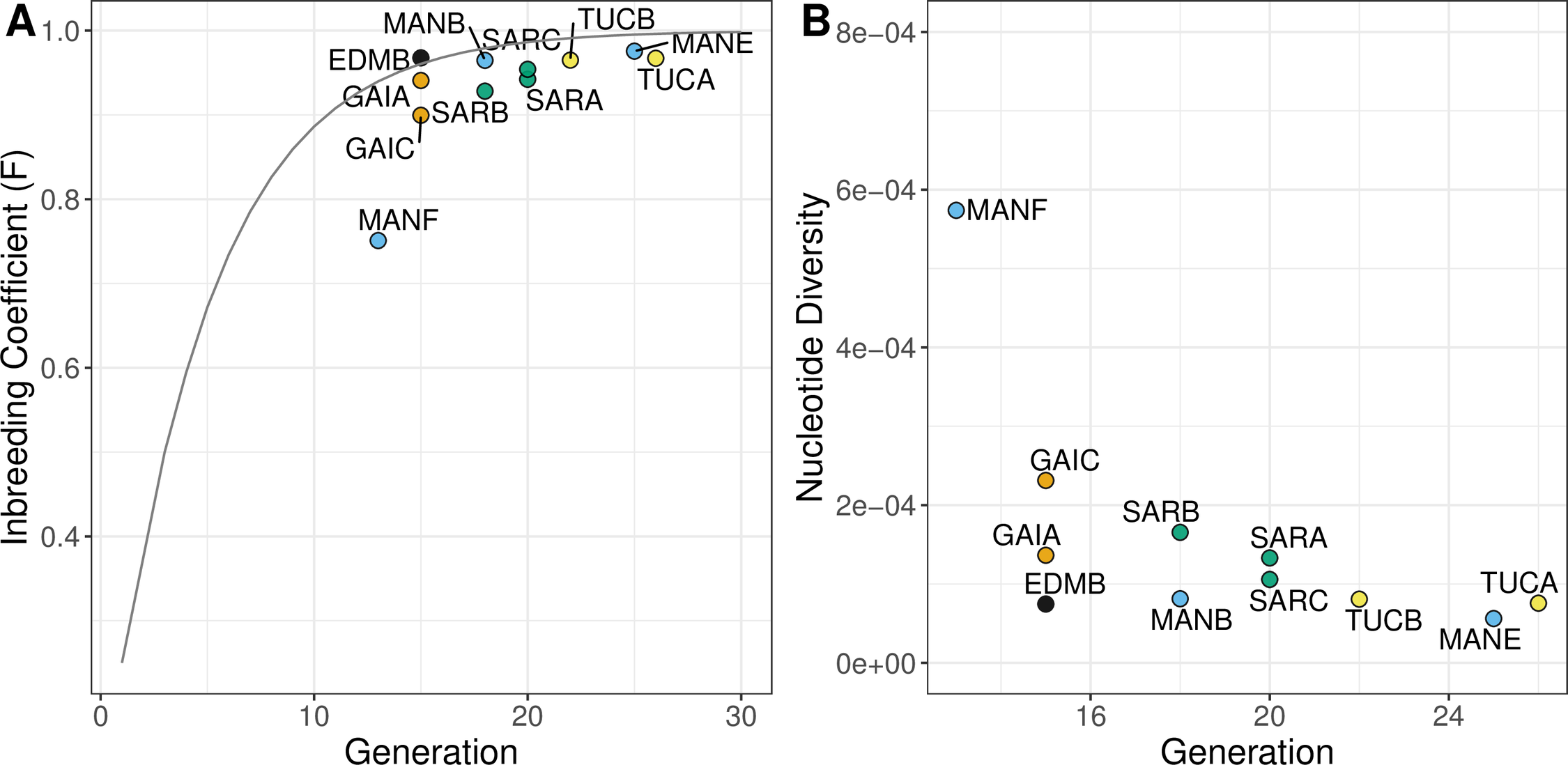

Supplement: S3 Fig — (A) The observed inbreeding coefficient, F, was calculated using the method of moments estimator implemented in vcftools (v 0.1.16) [11,90–92]. For most strains, the empirical F estimate closely tracks with the theoretical expectation for increasing inbreeding generation number (black line). This expectation was computed from the recurrence equation: Ft=0.25(1+2Ft−1+Ft−2), where Ft corresponds to the inbreeding coefficient at generation t. Strain MANF/NachJ presents an exception that is likely attributable to departures from a strict sib-sib mating design during the colony expansion effort that preceded strain rederivation. (B) Per sample nucleotide diversity (π) declines with increasing inbreeding generation, as expected. (TIF) [file pgen.1011228.s003.tif]

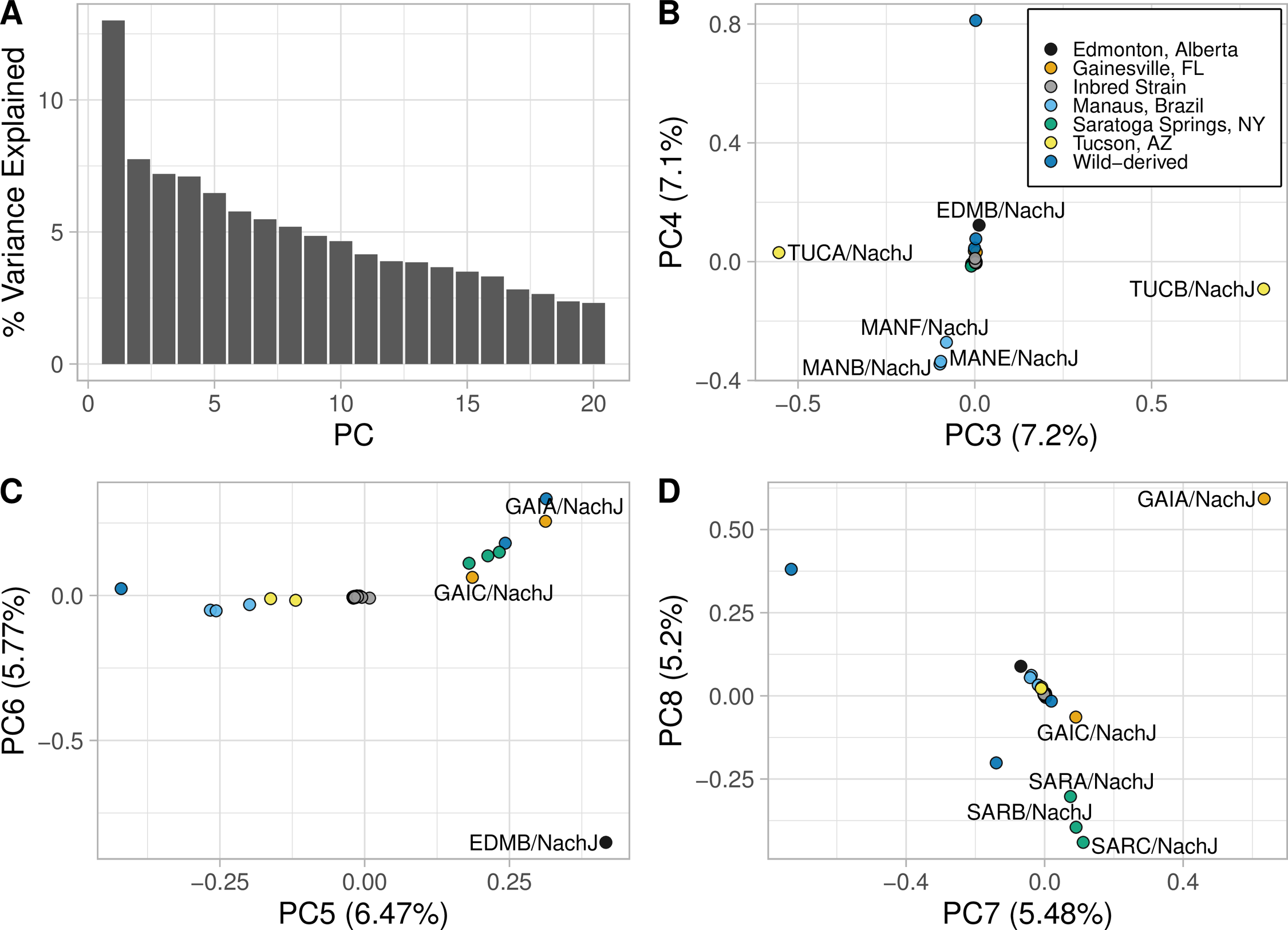

Supplement: S4 Fig — (A) Percentage of total variance explained by each PC 1–20. (B) Dot plots of PC3 versus PC4, PC5 versus PC6 (C), and PC7 versus PC8 (D). The color legend in (B) also applies to (C) and (D). (TIF) [file pgen.1011228.s004.tif]

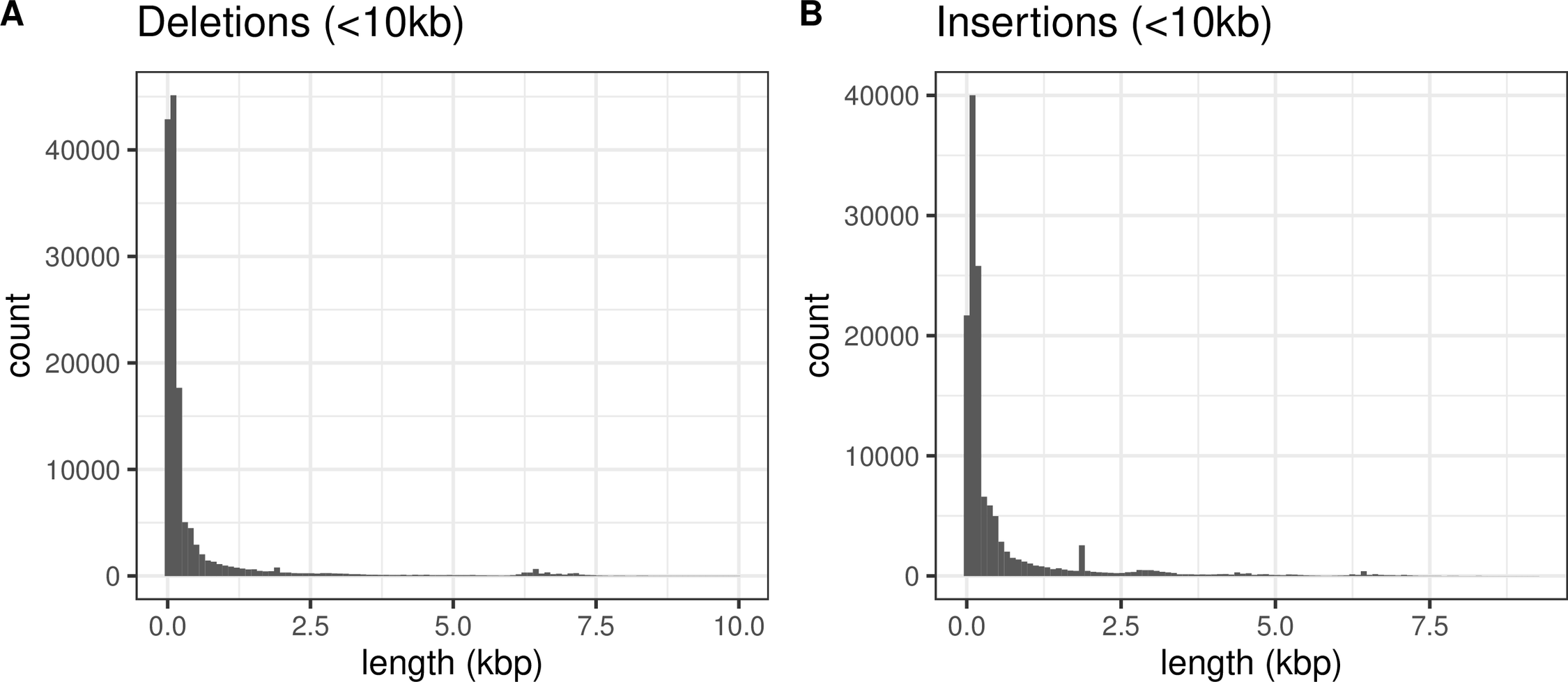

Supplement: S5 Fig — Distribution of deletion (A) and insertion (B) lengths across the Nachman strains. For ease of visualization, only SVs <10kb in length are plotted. Only 49 deletions and 1 insertion call exceed this cutoff. Peaks at ~200bp and ~6kb suggest contributions from SINE elements and full-length L1 elements. (TIF) [file pgen.1011228.s005.tif]

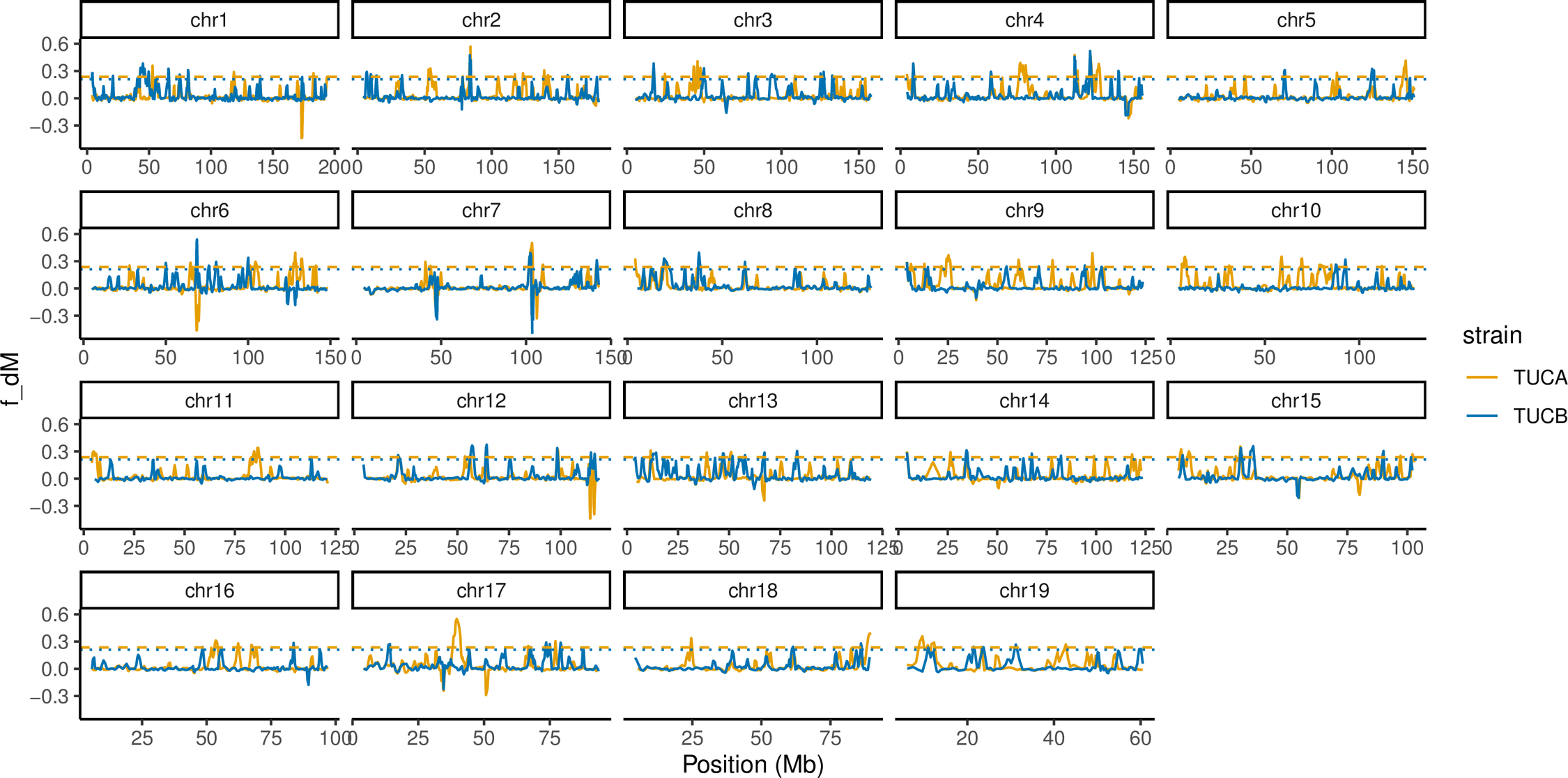

Supplement: S6 Fig — fdM was calculated in 5000 SNP windows (2500 SNPs slide) using genome sequences from wild-caught M. m. domesticus, wild-caught M. m. castaneus, and M. spretus (S8 Table). Dashed lines correspond to the 95th percentile of the most extremely positive fdM statistics for each strain, delimiting regions of likely M. m. castaneus introgression. (TIF) [file pgen.1011228.s006.tif]

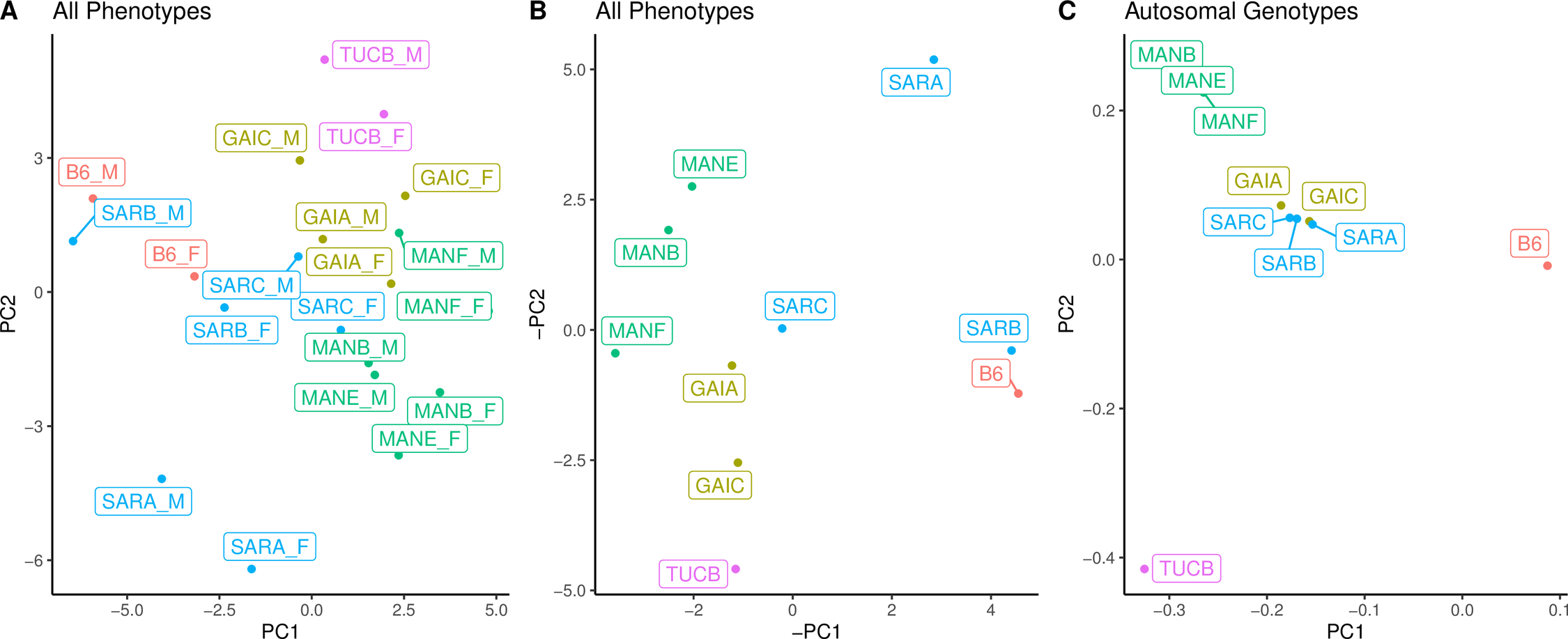

Supplement: S7 Fig — Individual PC analyses were first performed on normalized per sex, per strain mean trait values from each phenotyping assay. The minimum number of PCs required to explain >90% of the variance for each phenotype assay were aggregated, and a second PCA was performed on the resulting matrix. Results from this second PCA are plotted in (A), with PC1 (14.5%) and PC2 (11.9%) together accounting for 26.4% of the variance. PC values were averaged over sexes within a strain and axes rotated 180° to produce (B). Panel (C) duplicates the results from the genotypic PC analysis presented in Fig 3B to enable appreciation of the similarity of the strain distribution pattern along the first two PCs. (TIF) [file pgen.1011228.s007.tif]

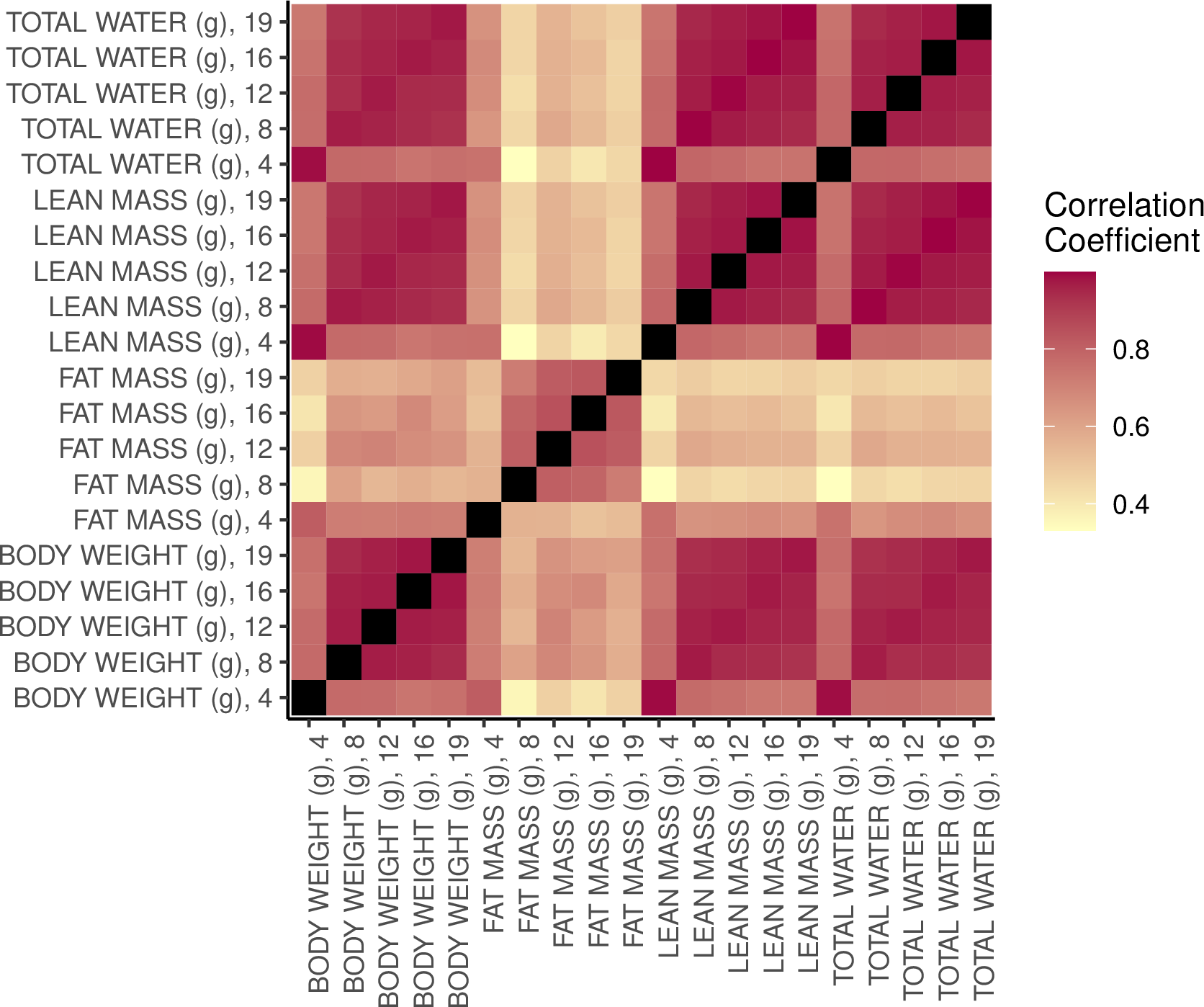

Supplement: S8 Fig — Numbers in the axis tick labels correspond to animal age at the time of measurement. The magnitude of the correlation coefficient (Spearman’s rho) is indicated by the scale bar, with higher correlations denoted by darker red colors, and weaker correlations indicated in yellow. (TIF) [file pgen.1011228.s008.tif]

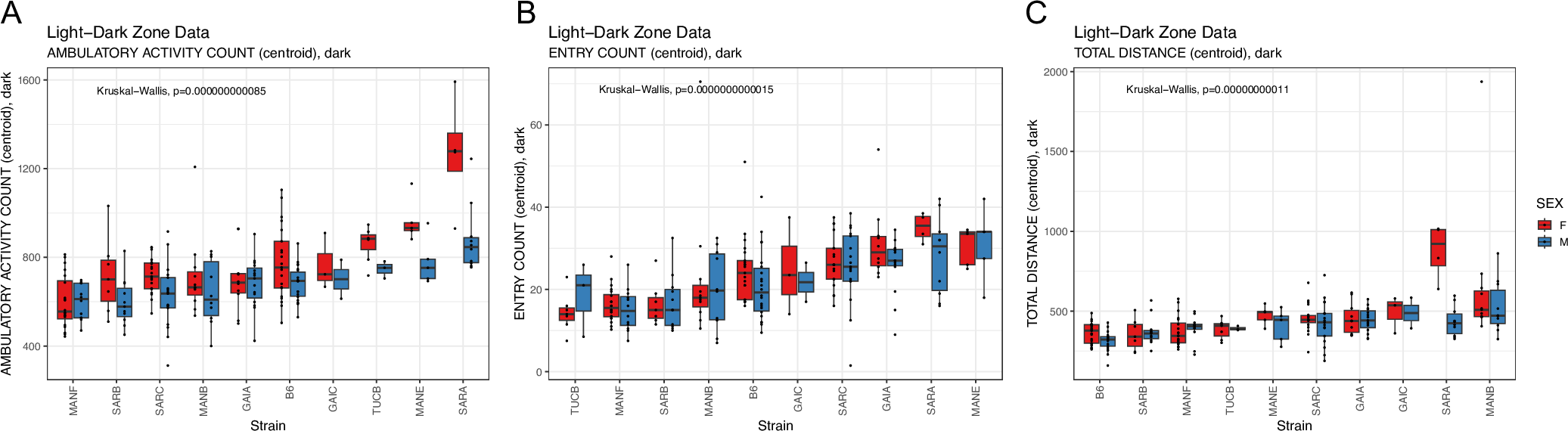

Supplement: S9 Fig — The centroid position on the test subject’s body was used to assign position in either the dark or light halves of the testing apparatus. In (C), distance is plotted on the y-axis in meters. The color legend presented in (C) applies to all panels. (TIF) [file pgen.1011228.s009.tif]

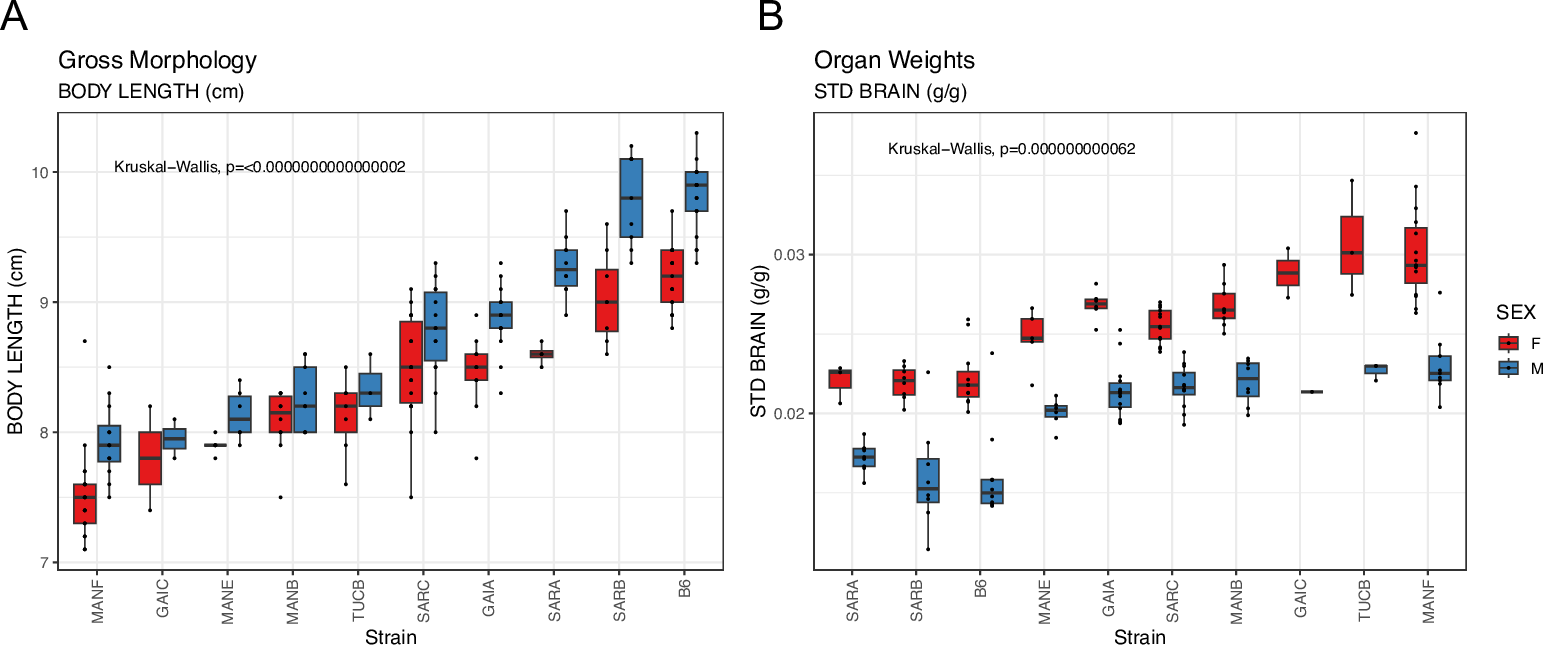

Supplement: S10 Fig — Strain and sex variation in body length (A) and (B) brain weight. Brain weight is standardized by total body weight. (TIF) [file pgen.1011228.s010.tif]

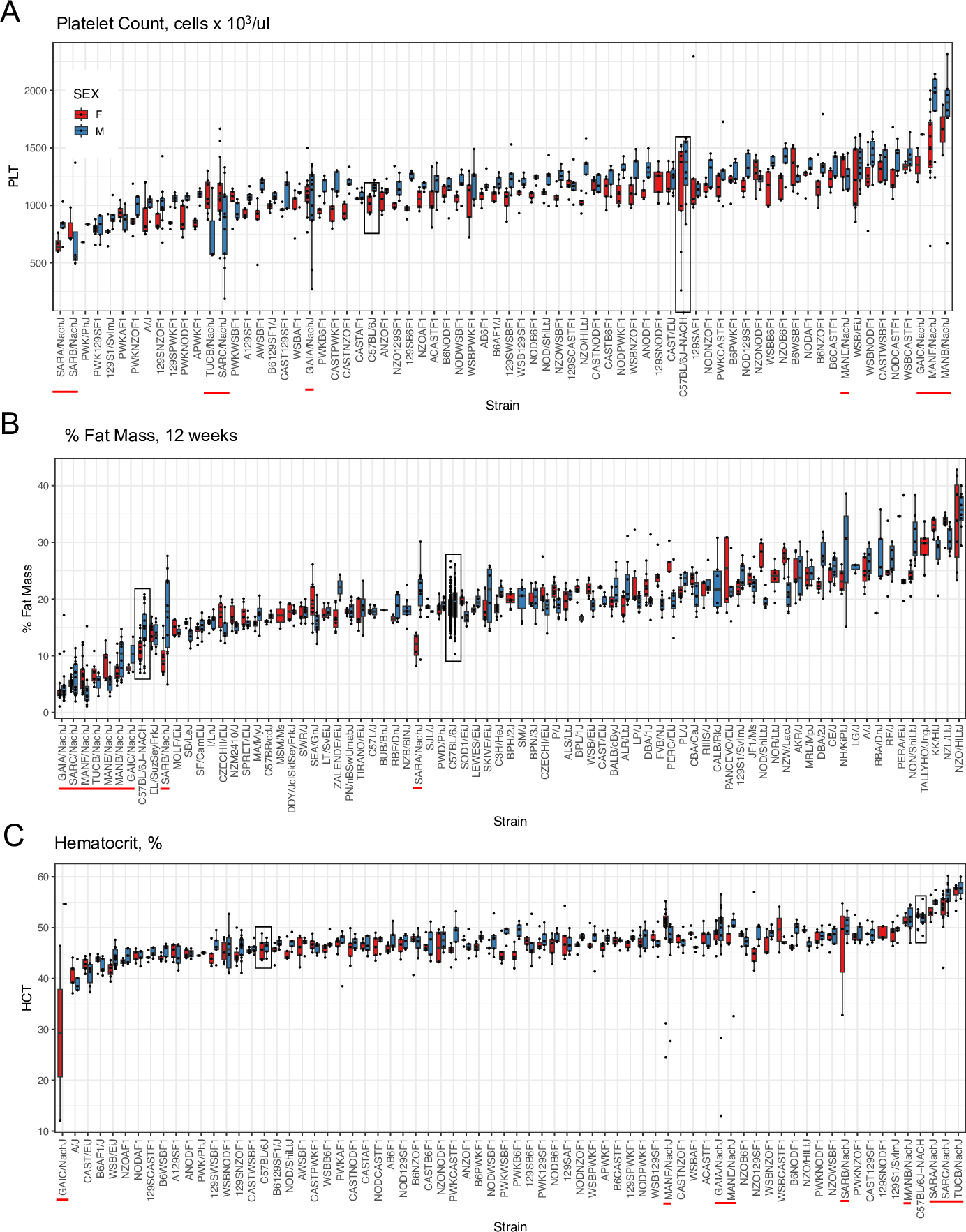

Supplement: S11 Fig — (A) Platelet counts (cells x 103/ul) for the Nachman strains were integrated with the CGDpheno3 dataset from the Mouse Phenome Database. (B) Percent fat mass at 12 weeks in the Nachman lines and strains profiled in the CGDpheno1 dataset. (C) Percent hematocrit in the Nachman lines and strains in CGDpheno3. The color legend in (A) applies to all panels. In each panel, Nachman strains are indicated by red bars along the x-axis and boxplots for C57BL/6J mice are marked by black boxes. C57BL/6J mice phenotyped as controls alongside the Nachman strains are denoted as “C57BL/6J-NACH”; C57BL/6J animals phenotyped in other studies are indicated by the x-axis label “C57BL/6J”. (TIF) [file pgen.1011228.s011.tif]

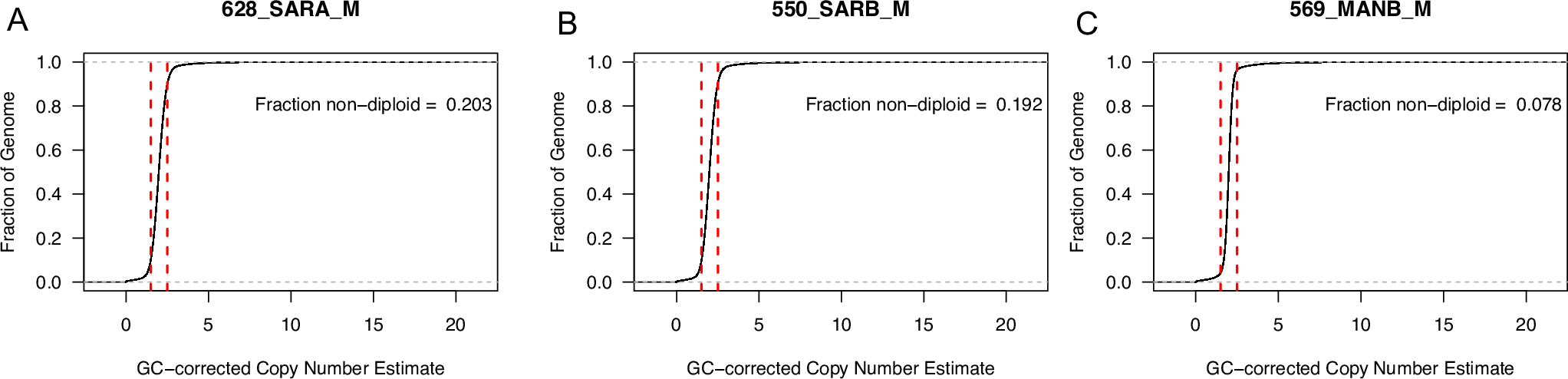

Supplement: S12 Fig — Cumulative distribution of copy number state in 1kb sliding windows across the autosomal genome for (A) SARA/NachJ, (B) SARB/NachJ, and (C) MANB/NachJ. To estimate copy number, read depth was first computed in 1kb sliding windows (no overlap) using mosdepth (executed command: mosdepth -n -b 1000 -t 2 -x $PREFIX $BAM). Raw read depth values were then corrected for potential GC-biases introduced during library preparation. Briefly, we used the GRCm39 reference genome to compute the observed GC content of each 1000bp window. GC content values were rounded to the nearest 0.001 and regions with identical GC content were binned. For each strain, we then computed the mean read depth across all genomic windows that fell into each GC content bin. Next, we fitted a second degree polynomial to the relationship between read depth and GC content using the scatter.smooth function in R and with span parameter of 0.7. For each GC-bin, we then computed the difference between the fitted polynomial and the genome-wide average read depth. These values correspond to the magnitude of “inflation” or “deflation” in read depth across windows of a given GC-content due to systematic GC biases in the data. The read depth value in each 1000bp window was then adjusted by the appropriate GC correction factor. Finally, these GC-corrected read depths were divided by the average per-sample coverage to convert into absolute copy number estimates. The cumulative distribution of autosomal copy number estimates was then computed using the ecdf function call within the stats package in Rstudio (version 2022.02.0, Build 443). The proportion of windows with copy number <1.5 and >2.5 (red vertical lines) was calculated as a proxy for the extent of structural divergence between the focal strain genome and GRCm39. (TIF) [file pgen.1011228.s012.tif]
