# Supplementary material for: Into the Wild: A novel wild-derived inbred strain resource expands the genomic and phenotypic diversity of laboratory mouse models
Source: PLoS Genet. 2024 Apr 10;20(4):e1011228. doi: 10.1371/journal.pgen.1011228 (PMC11034653; doi:10.1371/journal.pgen.1011228)
Supplement: Attachment — Submitted filename: 2024_0229_Reviewer_Responses.docx [file pgen.1011228.s046.docx]

**Reviewer #1: This is a remarkably well written paper introducing the wild derived inbred strain panel (the Nachman panel). The authors have done a tremendous amount of work characterizing the genomic and phenotypic diversity of this panel. An import feature that sets these strains apart from classical inbred mouse strains is the absence of artificially directed selection (selection on fitness will nonetheless occur), which make genetic variation in this panel more resembling human populations, thus presenting better models for human traits. In all aspects surveyed, there is substantial genetic variation, making it an attractive resource, especially considering the deep phenotyping across ages as well as planned phenotyping. I recommend publication as is, but here are a few points for the authors to consider during revision. I would like to emphasize that the paper in its present form is outstanding and deserves to be published.**

We thank the reviewer for their constructive feedback on our paper and are encouraged by their laudatory remarks.

**1. It may be helpful to perform a PCA based on the large collection of phenotypes as a way to separate the lines based on phenotypes and compare the separation pattern with that based on genotypes.**

We first performed PC analyses on phenotypes from each individual assay. In our judgement, the resulting PC plots provide little new biological insight not already revealed by other analyses in the paper (**Fig 1**). For instance, B6, SARA, and SARB separate from other strains for organ weights and (to a lesser extent) the NMR assay, reflecting the overall higher body mass of these strains. This result is revealed in Fig 7 of the main manuscript. For many phenotypes (e.g., clinical chemistry, glucose tolerance test, NMR), strains loosely cluster by geographic origin, recapitulating findings from linear modeling (Supplementary Tables 21-23; lines 426-434). For a given strain, males and females are often loosely clustered along the first two PCs, echoing results from the model-based analyses presented in Supplementary Tables 18 and 19.

**Fig 1.** Plots of PC1 versus PC2 for traits estimated in each phenotyping assay. Analyses were performed on normalized per sex, per strain mean trait values. Samples are color-coded based on geographic origin. Sample labels are presented in the format: {Strain}_{Sex: M or F}.

We then combined the minimum number of PCs required to explain >90% of the variance for each phenotype assay and performed a PCA on the resulting matrix (**Fig 2A**). The resulting plot exposes loose clusters based on strain geographic origin, reaffirming results from analyses presented in Supplementary Tables 21-23. We have included this plot as a supplemental figure in the revised manuscript (Supplementary Figure 7). This new figure is referenced on lines 439 and 442, and the methodology is described on lines 1188-1198.

**Fig 2.** Comparison of phenotypic and genotypic PC analysis results. (A) PC analysis on all phenotypes. PC1 (14.5%) and PC2 (11.9%) together account for 26.4% of the variance. (B) The phenotypic PC plot in (A) rotated 180° about the origin to enhance visual correspondence with the genotypic PC plot shown in (C). Strains are color-coded by geographic origin.

We note that there is some spatial correspondence between the strain distribution pattern observed in the phenotypic PCA and that seen in the genotypic PCA (**Fig 2C**). To visually appreciate this similarity, we rotated the phenotype plot 180° about the origin (i.e., reversed the sign of both PC1 and PC2; **Fig 2B**) and averaged the PC values for the two sexes of a given strain. On both plots, PC1 partitions B6 to the right, with Saratoga strains closest to B6 along this dimension. PC2 isolates TUCB away from other strains in both phenotypic and genotypic space. Manaus strains cluster in the upper left region of both plots. This visual similarity is supported by a strong positive correlation between the sex-averaged phenotypic and genotypic PC matrices (Adjusted RV = 0.471) and a significant similarity of matrices index (SMI = 0.709, P = 0). Overall, the general agreement between the two PC plots is consistent with polygenic control of the surveyed phenotypes that inform the phenotypic PCA. We have reported these findings in the revised manuscript (lines: 439-443).

**2. When annotating variants using VEP, is the phasing between sites taken into account? For example, a frameshift indel can be shifted back in frame by another frameshift indel. If they are considered separately, they may lead to two loss of function events when the protein is in fact functional.**

VEP does not explicitly account for multiple indels in a gene. However, we do not think that this consideration is likely to over-inflate our assessment of the functional impact of variants present in the Nachman strains. First, we exclude short indels and deletions from our analyses, instead focusing on SNPs and larger-scale structural variants (>50bp). SNPs, by definition, will not alter the reading frame. Second, virtually all SVs annotated as deleterious are predicted to impact splicing, ablate transcripts or regulatory regions, or activate nonsense-mediated decay. It is unlikely that a second, linked SVs would counteract these impacts.

**3. The PCA analysis integrating the Nachman strains and other strains needs to be cautious because the variants are called using different technologies, introducing a batch effect.**

We have added an acknowledgment of this important caveat in the revised manuscript (page 11, Lines 251-254):

*However, we acknowledge that differences in the technologies used to sequence the Nachman strains (PacBio HiFi) and other strains (Illumina) could partially contribute to the isolation of the Nachman strains* along these PC axes*.*

**4. The value of this resource lies at not only the mouse colonies but also the genotype and phenotype data. How will these be accessible to the public? At this time raw data are deposited and other data are provided as supplemental tables, which aren't ideal. Please note that this is beyond the scope of the paper and the authors probably have a plan already. But I think some forward looking discussion in this paper will go a long way.**

We fully agree and share the reviewer’s enthusiasm for data sharing. All collected phenotype data are available in the supplemental materials accompanying this manuscript. In particular, Supplementary Table 24 includes all measured phenotype values in long-data format for ease of analysis in R. In addition, we have provided all phenotype data to curation staff at Mouse Phenome Database; data are currently “In Review” by MPD curation staff. Variant call datasets are available for download on Zenodo (10.5281/zenodo.10728008).

We have added the following sentence to the paper to point readers to these resources (lines 824-827). Additional information about how to access these resources is provided in the *Data Availability* statement.

*Recognizing that the scientific value of this strain resource is directly tied to the availability of genomic and phenotypic resources, we have made all raw (e.g., fastq files, raw phenotype values) and derived data (e.g., vcf files, strain phenotype means) presented in this manuscript available in public repositories and as supplemental material.*

**5. Can the authors provide some hypothetical examples of how one might use this resource? It was mentioned that crossing these lines to CIs, QTL mapping, etc. But those do not have to utilize these strains.**

We acknowledge that there are other mouse diversity resources investigators can use. In our judgement, the single most important innovation presented by the Nachman strain resource *“is its exclusive profile of the natural genetic and phenotypic variability in* M. musculus*”* (lines 735-736). As we emphasize on lines 736-744, the absence of overt selection for reproductive output, ease of handling, and other traits of interests during the generation of these strains represents a notable contrast to the origin history of classical inbred (CI) mouse strains. As a result, strains in the Nachman panel are poised to better approximate the diversity and organization of functional genetic variation in human populations, potentially increasing the translatability of preclinical findings from mouse to human. Use of these strains in place of traditional CI lines is, on its own, likely to lead to new biological discoveries, regardless of the experimental paradigm pursued.

In addition to these arguments, our original submission presented several possible use cases for this resource. For example, on lines 725-730, we suggest that these strains could be used in conventional genetic mapping studies:

*The Nachman strain panel complements existing diverse mouse platforms, such as the BXD (62), CC (22), HMDP (63), and DO (23), offering a new community resource for profiling phenotypic variation and assessing responses to exposures, interventions, or treatments. While the 11-member panel is too small to power genetic mapping studies, experimental crosses between Nachman strains with extreme phenotypes can be employed to generate F2, backcross, or advanced intercross lines for experimental mapping.*

We have added the following sentence to this paragraph to highlight an additional experimental use case for this resource (lines 730-732):

*Similarly, crossing knock-out alleles into the Nachman strains could enable the identification of naturally segregating modifiers of disease penetrance, severity, or onset.*

On lines 779-781, we suggest that the Nachman strains could be used to assess the range of responses to various experimental perturbations, exposures, or treatments:

*Experimental designs that employ environmental perturbations, different exposure or treatment regimes, aged animals, or other deliberate manipulations may uncover novel phenotypic responses or resilience/susceptibility traits.*

We also reference our intention of generating a new outbred population from a subset of these inbred strains (lines 827-830). It is envisioned that this future resource will enjoy broad use in genetic mapping studies and tests of organismal response to exposures, treatments, and various experimental manipulations. We have also added mention of planned future efforts to create embryonic stem cell lines from these strains. Such a resource will provide a platform for cell-based assays and germline genetic engineering.

*Our team is currently engaged in efforts to expand the collection of tools and resources presented here, including de novo genome reference assemblies,* embryonic stem cell lines, *gene expression datasets, and the initiation of an outbred population founded from a subset of 4 Nachman strains.*

While these use case scenarios are not exhaustive, we believe that they are encompassing of the most common experimental paradigms that stand to benefit from adoption of the Nachman panel.
